# Prevalence of Coronary Artery Disease Evaluated by Coronary CT Angiography in Women with Mammographically Detected Breast Arterial Calcifications

**DOI:** 10.1371/journal.pone.0122289

**Published:** 2015-04-09

**Authors:** Leila Mostafavi, Wanda Marfori, Cesar Arellano, Alessia Tognolini, William Speier, Ali Adibi, Stefan G. Ruehm

**Affiliations:** 1 Department of Radiological Sciences, David Geffen School of Medicine, University of California Los Angeles, Los Angeles, CA, United States of America; 2 Department of Radiological Sciences, University of California Irvine, Irvine, CA, United States of America; 3 Medical Imaging Informatics, Department of Radiological Sciences, University of California Los Angeles, Los Angeles, CA, United States of America; University of Miami, UNITED STATES

## Abstract

To assess the correlation between breast arterial calcifications (BAC) on digital mammography and the extent of coronary artery disease (CAD) diagnosed with dual source coronary computed tomography angiography (CTA) in a population of women both symptomatic and asymptomatic for coronary artery disease. 100 consecutive women (aged 34 – 86 years) who underwent both coronary CTA and digital mammography were included in the study. Health records were reviewed to determine the presence of cardiovascular risk factors such as hypertension, hyperlipidemia, diabetes mellitus, and smoking. Digital mammograms were reviewed for the presence and degree of BAC, graded in terms of severity and extent. Coronary CTAs were reviewed for CAD, graded based on the extent of calcified and non-calcified plaque, and the degree of major vessel stenosis. A four point grading scale was used for both coronary CTA and mammography. The overall prevalence of positive BAC and CAD in the studied population were 12% and 29%, respectively. Ten of the 12 patients with moderate or advanced BAC on mammography demonstrated moderate to severe CAD as determined by coronary CTA. For all women, the positive predictive value of BAC for CAD was 0.83 and the negative predictive value was 0.78. The presence of BAC on mammography appears to correlate with CAD as determined by coronary CTA (Spearman’s rank correlation coefficient = 0.48, p<.000001). Using logistic regression, the inclusion of BAC as a feature in CAD predication significantly increased classification results (p=0.04).

## Introduction

Coronary artery disease is the leading cause of mortality in both genders in the U.S [1]. The rate of coronary artery disease (CAD) related mortality has declined in men but has not significantly changed in women [2]. Overall, more American women than men die of CAD annually, and until the age of 80 years, women who suffer from acute coronary syndrome suffer higher mortality rates [1, 2, 3]. Various causes postulated to account for the difference in CAD-related mortality include decreased disease detection in women and gender differences in symptomatology and pathophysiology [2, 3]. Early detection and treatment of coronary artery disease remain underutilized among women.

A few prior studies have attempted to establish a relationship between breast arterial calcification (BAC) and CAD risk factors. The majority of these studies demonstrated a positive association between BAC and increasing age. However, the relationship between BAC and CAD risk factors such as diabetes mellitus, renal disease, hypertension, hyperlipidemia, and obesity remains inconclusive [4–8]. Surprisingly, a persistent inverse association appears to exist between BAC and smoking [4–6, 9, 10]. Additional studies have also demonstrated a positive association between BAC and lactation as well as parity [5, 11, 12]. Large scale cohort studies and retrospective studies have almost uniformly suggested a strong association between BAC and cardiovascular disease related morbidity and mortality [4, 6, 9, 10, 13–16]. This strong association of BAC with cardiovascular pathology suggests that BAC should also be persistently associated with radiographically determined CAD.

The gold standard of CAD determination has historically been catheter coronary angiography. Prior studies that attempted to establish a relationship between BAC and CAD as defined by coronary angiography have produced inconsistent results: two studies reported a positive association [17–19]; two studies reported an indeterminate association [7, 17]; and two studies reported a negative association between BAC and CAD as determined by catheter angiography [16, 20].

Screening mammography is a widely used diagnostic test to detect breast cancer. If BAC could serve as a marker for CAD, then screening mammography could yield added value as a single test already in wide use for the additional detection of CAD, an additional highly prevalent disease with high morbidity and mortality.

Dual source coronary computed tomography angiography (CTA) has been shown to be effective for non-invasive diagnosis of CAD based on the demonstration of calcified and non-calcified coronary arterial plaque and grading of stenotic disease [21]. To the best of our knowledge, no study has described the association between BAC and CAD as diagnosed by coronary CTA.

This study was conducted to assess the relationship between mammographic BAC and CAD based on coronary CTA findings. Medical records for 100 patients who received both a mammogram and a coronary CTA were reviewed for imaging findings and CAD risk factors. Correlation was measured between BAC and CAD and logistic regression was used to create a classifier for predicting CAD based on BAC and common risk factors.

## Materials and Methods

This Health Insurance Portability and Accountability Act (HIPAA) compliant study was approved by the local institutional review board and the informed consent requirement was waived.

### Data Collection

The study population comprised 100 consecutive women (aged 34–86 years; mean, 65.3 years; median, 65.5 years) who underwent both coronary CTA and digital mammography at our institution between August 2008 and December 2012. Neither coronary CTA nor digital mammography was conducted for study purposes. Both studies were obtained within six months of each other and the order varied between patients (mammogram was first for 48/100 patients). The study population consisted of all patients who received both of these tests at our institution between these dates and the studies were identified by computer generated lists. Twelve women underwent coronary CTA as part of a general health screening protocol and were asymptomatic for coronary artery CAD. The remaining 88 women were symptomatic and underwent coronary CTA for diagnostic purposes based on an at least low probability for CAD. For those patients with more than one set of mammograms and/or coronary CTA, the most recent set of exams was evaluated. Applicable coronary CTA examinations performed between August 2008 and December 2012 were included in the study. The corresponding mammograms were performed between April 2008 and December 2012.

**Fig 1:**
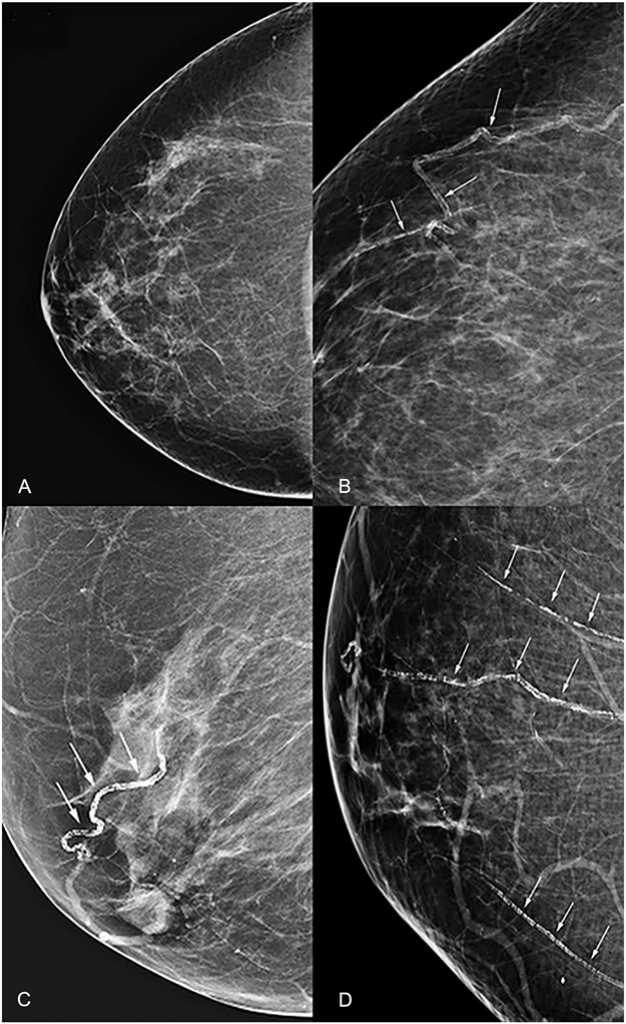
Breast arterial calcification (BAC) scoring on mammography. A. Grade 1: No vascular calcifications. B. Grade 2: Few punctate vascular calcifications, no tram track or ring calcifications. C. Grade 3: Coarse or tram track calcifications affecting <3 vessels. D. Grade 4: Coarse or tram track calcifications affecting ≥3 vessels.

The mammograms were obtained for screening (n = 88) and diagnostic purpose (12) on a Hologic Selenia digital mammography unit (Hologic, Inc., Bedford, MA, USA). Two physicians (A.T., L.M.) independently reviewed each mammogram on a PACS system (General Electric, Milwaukee) to grade the degree of breast arterial calcifications (BAC) using a four point scale, considering severity and extent of calcifications: 1: No vascular calcifications; 2: Few punctate vascular calcifications, no areas of tram track (2 parallel lines) or ring calcifications, 3: Coarse vascular calcifications of definite tram track or ring appearance affecting fewer than three vessels; and 4: Severe coarse vascular calcifications affecting three or more vessels (Fig 1). Grade 3 (moderate) and 4 (severe) vascular calcifications were regarded as positive BAC for additional statistical analyses.

**Fig 2:**
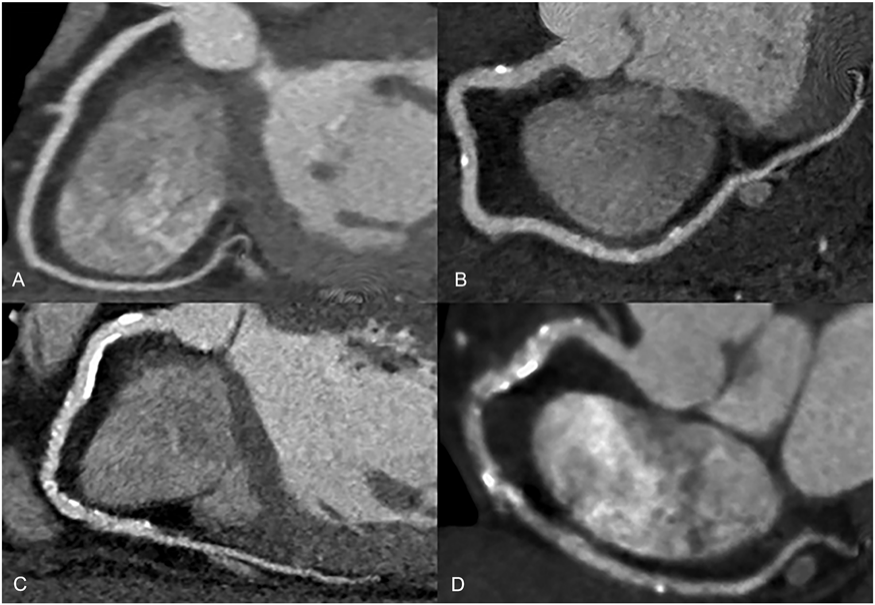
Coronary artery disease (CAD) scoring on CTA. A. Grade 1: No coronary artery disease. B. Grade 2: Eccentric calcifications with mild atherosclerotic irregularities. C. Grade 3: Scattered calcifications, stenoses <50%. D. Grade 4: Vascular calcifications and stenosis ≥50%.

Coronary CT angiograms were obtained on a Somatom Definition Dual Source CT scanner (Siemens Healthcare, Erlangen, Germany) following a routine clinical protocol using 80–100 mL of iodinated contrast agent (Isovue 370, Bracco, Milan, Italy). Two physicians (S.R., C.A.) independently reviewed each coronary CT angiogram to grade the severity of CAD. CT data sets were available on a PACS and an image processing workstation (Vital Images Inc. Minnetonka, Minnesota), which enabled interactive reconstruction of multiplanar and curved reformatted data sets and volume rendered views. A CAD score was assigned according to a four point scale, graded by the extent of calcified and non-calcified plaque and the degree of major vessel stenosis: 1: No coronary artery disease (disease defined as arterial calcium, plaque, major vessel stenosis, or irregularity); 2: Single or scattered eccentric arterial calcifications or vessel stenosis ≤10%; 3: Calcified or non-calcified arterial plaque with vessel stenosis <50%; and 4: Calcified or non-calcified plaque with vessel stenosis ≥50% occlusion, including patients who received a coronary stent or coronary artery bypass surgery for revascularization of at least one coronary artery (Fig 2). Grade 3 (moderate) and 4 (severe) disease were considered positive CAD.

Electronic medical records were reviewed for the following cardiovascular risk factors: hypertension (HTN), hyperlipidemia, diabetes mellitus, smoking, known atherosclerotic cardiovascular disease (ASCVD), and family history of ASCVD (Table 1). Hypertension was defined as a reported history or diagnosis of HTN, the use of anti-hypertensive drugs, or at least two measurements of systolic arterial blood pressure >140 and/or diastolic blood pressure >90 mmHg. Hyperlipidemia was defined as a lipid panel consistent with hyperlipidemia or the use of a lipid-lowering agent for at least two years. Diabetes mellitus was defined as the regular use of insulin or oral diabetic agent (e.g. Metformin). Any reported history of smoking within the 12 months prior and up to the time of the mammogram was categorized as positive. ASCVD was defined as a history of angina, myocardial infarct, abnormal catheter angiogram of coronary, carotid, abdomino-pelvic or lower extremity vascular territories, coronary artery bypass graft surgery (CABG) or stroke. A positive family history of ASCVD was defined as a first degree relative who had a cardiac event before the age of 55 if male, or before the age of 65 if female (cardiac events include angina, myocardial infarction (MI), abnormal coronary angiogram, CABG, stroke).

**Table 1:** Subject Characteristics.

		Count	(%)
**Total**		100	(100)
**Age (median)**		65.5	
**Hypertension**		64	(64)
**Hyperlipidemia**		57	(57)
**Diabetes Mellitus**		20	(20)
**Smoking History**		8	(8)
**Atherosclerotic Cardiovascular Disease**		60	(60)
**Family History**		43	(43)
**BAC:**	**1**	80	(80)
	**2**	8	(8)
	**3**	10	(10)
	**4**	2	(2)
**CAD:**	**1**	45	(45)
	**2**	26	(26)
	**3**	19	(19)
	**4**	10	(10)

CAD: Coronary Artery Disease, BAC: Breast arterial calcification

### Statistical Analysis

For grading of CAD on CT angiograms and BAC on mammograms a four point scale was used as described above. In cases of discrepancies of grading between the two readers, a senior cardiac/breast radiologist with more than 15 years of imaging experience was consulted to determine final grading which was consequently used for statistical analysis. Additionally, both BAC and CAD were redefined as binary variables by assigning a negative value to grades 1 and 2 (no disease and mild, respectively) and a positive value to grades 3 and 4 (moderate and severe, respectively).

Cohen’s kappa coefficients were used to measure inter-observer agreement. The correlation between BAC and CAD was calculated using Spearman’s rank correlation coefficients and significance was determined using a permutation test. Patients were then stratified by age, determined by whether their age was greater than the overall median. To show the effect of age on the relationship between BAC and CAD, additional Spearman’s rank correlation coefficients were determined for each of the age groups.

Logistic regression was used to attempt to predict the presence of CAD based on sets of patients’ risk factors: age only, age and BAC, and all risk factors. Using each set of risk factors, leave-one-out cross-validation was employed to determine a classifier and then attempt to predict the presence of CAD for each patient. Receiver operator characteristic (ROC) curves were then plotted and the area under the curve (AUC) was determined for each set of risk factors. Wilcoxon rank-sum tests were performed on each of the ROC curves to test for significant performance above random chance and differences between results using different sets of risk factors were tested using a modified sign test [22].

## Results

**Fig 3:**
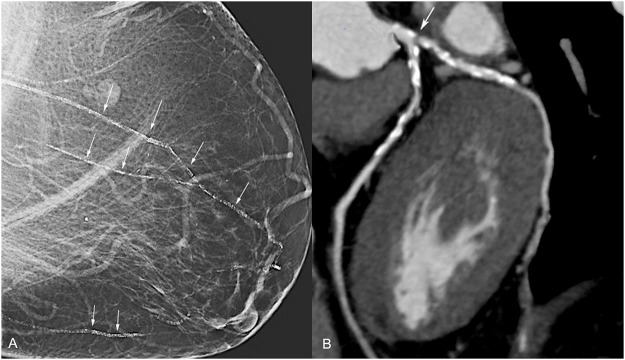
Images from a patient with mammography and CT angiography findings. Screening mammography in 65-year-old patient demonstrates severe vascular calcifications (Grade 4) on screening mammogram (A). Corresponding coronary CT angiogram (B) shows diffusely calcified proximal left anterior descending and left circumflex artery with focal high-grade stenosis of (arrow).

Inter-observer variability scores for BAC (kappa: 0.86) and CAD (kappa 0.67) were excellent and good, respectively. The overall prevalence of positive BAC in the studied population was 12% (12/100) and the overall prevalence of CAD was 29% (29/100). The positive predictive value of BAC for CAD was 0.83 (10/12 patients) while the negative predictive value was 0.78 (69/88 patients). The specificity of BAC for CAD was 0.97, while the sensitivity of BAC for CAD was 0.34. Fig 3 demonstrates a patient with high grade BAC and CAD.

Using a four scale scoring system, there was a statistically significant positive association between BAC and CAD (Spearman’s correlation coefficient = 0.48, p<0.001) (Table 2). Stratifying the patients based on age resulted in two subgroups of size 50: those older than the median age (65.5) and those younger. Both the old (Spearman’s correlation coefficient = 0.39, p = 0.003) and the young (Spearman’s correlation coefficient = 0.38, p = 0.007) subgroups showed associations between BAC and CAD that were statistically significant, but weaker than the overall association.

**Table 2:** Frequency plot for Coronary Artery Disease (CAD) and Breast Arterial Calcification (BAC).

		CAD			
		1	2	3	4
**BAC**	**1**	44	19	14	3
	**2**	1	5	2	0
	**3**	0	2	2	6
	**4**	0	0	1	1

**Fig 4:**
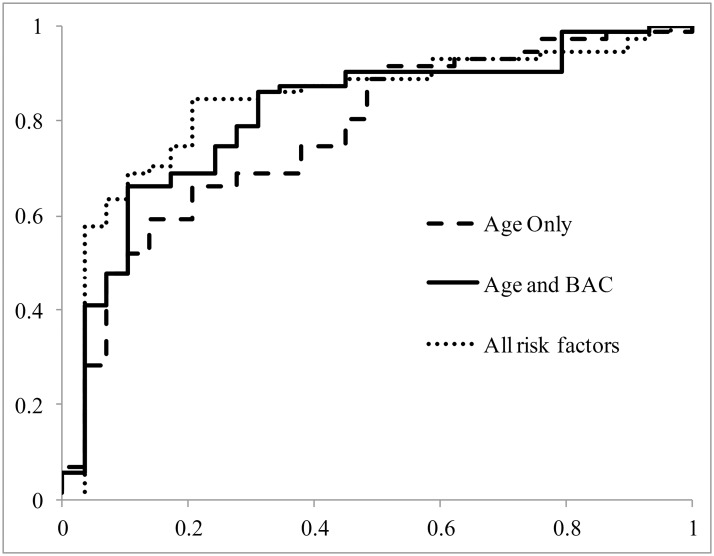
Receiver Operator Characteristic (ROC) Curves for coronary artery disease prediction. ROC curves plotted for the results of leave-one-out cross validation using logistic regression with only age (dashed), age and CAD (solid), and all variables (dotted) included in classification. Including CAD in classification yields significantly better results (p = 0.04) than only classifying on age. Including all variables yielded the highest area under the curve, but was not significantly better than classifying with only age and CAD (p = 0.28).

Using logistic regression, classifiers for predicting CAD were created using three groups of risk factors: age only, age and BAC, and the full set of risk factors (Fig 4). All three classifiers predicted CAD significantly better than random (p<0.001, p<0.001, and p<0.001, respectively) (Table 3). When predicting based only on age, the area under the ROC curve was 0.77. Adding BAC as a feature in classification increased the AUC significantly to 0.81 (p = 0.04). Using all risk factors in classification produced the best AUC of 0.83, but the result was not significantly different from predicting based only on age and BAC (p = 0.28).

**Table 3:** AUC and significance values for results of classification of CAD using different sets of risk factors.

Risk Factors	AUC	Significance
**Age**	0.77	<0.001
**Age and BAC**	0.81	<0.001
**Age, BAC, Hypertension, Hyperlipidemia, Diabetes Mellitus, Smoking History, Atherosclerotic Cardiovascular Disease, and Family History**	0.84	<0.001

CAD: Coronary Artery Disease, BAC: Breast arterial calcification

## Discussion

This study suggests that presence of moderate to severe BAC on screening mammography is correlated with moderate to severe CAD as determined by coronary CTA (Spearman’s rank correlation coefficient = 0.48, p<0.001). This information is partially captured by the patient’s age as the correlation decreases after age stratification (Spearman’s rank correlation coefficients of 0.39 and 0.38 for patients older and younger than median, respectively), but not completely as the correlation is still significant (p = 0.003 and p = 0.007, respectively).

When predicting CAD based on risk factors, adding BAC significantly increased classifier AUC over using only age. This improvement demonstrates that BAC contains information useful in predicting CAD that is not captured by the patient’s age. The fact that classifying on all risk factors did not significantly increase the AUC indicates that much of the useful information for CAD prediction is provided by age and BAC. Although including other risk factors did not result in a significant improvement, it is expected that they provide some additional information that is useful in prediction. The lack of significance in this study might have been due to the small sample size.

Whereas catheter coronary angiography remains diagnostic standard for diagnosing CAD, it has limitations due to its projectional nature and is therefore less sensitive than cross-sectional coronary CTA for detection of early atherosclerotic changes. Of note, particularly non-calcified non-obstructive plaque has been linked to an elevated risk of cardiac events [23].

Our literature review yielded nine studies that have investigated the relationship between BAC and catheter coronary angiography, with four studies demonstrating a positive association [18], [19], [24], [25]; a mixed relationship was demonstrated in two studies [17], [7]. Three studies, however, disputed existence of this relationship [20], [16], [26], postulating that BAC has the strongest association with increasing age.

A study by Henkin et al showed that despite being associated with several risk factors for CAD, BAC did not distinguish patients with CAD from those with angiographically normal coronary arteries. One drawback of this study was the exclusion of patients with mild atherosclerosis on angiography, which likely contributed to underreporting CAD. The unexpected high prevalence of BAC in women without CAD (37%), compared to BAC in the CAD group (43.9%), may also have decreased the sensitivity of BAC as a marker for CAD. In contrast, the overall prevalence of BAC in our study population (12%) was consistent with the prevalence of BAC established in prior studies, which ranged from 6–23% [4, 12, 14, 15, 17, 18, 27–30].

A study by Zgheib et al did not demonstrate a correlation between BAC and CAD on coronary angiography. However, the calculation of the sample size of the study was based on a conservative estimation that about 12% of women older than 50 years have BAC; in addition the study suffered from a selection bias, since all subjects enrolled had prior diagnostic catheter coronary angiography and therefore likely presented symptoms suspicious for CAD [16]. This biased selection approach might have weakened the association between BAC and CAD. In contrast, twelve of our patients underwent coronary CTA as part of a screening protocol and were asymptomatic for coronary artery disease.

Several studies that compared the relationship of BAC to CAD as defined by modalities other than cardiac catheter angiography, however, have been more consistent in demonstrating a positive correlation. One prospective study conducted by Markopoulos et al [27], demonstrated a statistically significant association between BAC and color duplex ultrasound findings of atherosclerotic disease in the carotid or femoral arteries.

A small retrospective study evaluating the association of coronary artery calcifications identified on multislice CT and BAC demonstrated a strong, linear correlation [31]. A prospective study of 499 women demonstrated a strong association between BAC and CAD as determined by multislice CT Agatston calcium scoring [5]. While these findings are significant, they may in fact underestimate an association between BAC and CAD. While CT calcium scoring has been demonstrated to be an independent predictor of ASCVD risk, it does not account for the presence of non calcified plaque nor does it determine the degree of vessel stenosis. These studies which defined CAD by coronary calcium scoring or large vessel ultrasound are consistent with at least two large scale cohort studies that have demonstrated that BAC is associated with an increased risk of cardiac morbidity and mortality [10, 13–15].

Additional studies have demonstrated an association of BAC with ASCVD [4, 6, 9, 16, 32]. Interestingly, one study that failed to establish a relationship between BAC and catheter angiography, still demonstrated a strong positive association between BAC and cardiovascular morbidity and mortality [16].

Limitations of our study include a small sample size of 100 patients, the low prevalence of certain cardiac risk factors such as smoking, diabetes, and hyperlipidemia, an overall older patient population (age range: 34–86 years; mean: 65 years) and a selection bias since the majority of patients were referred for coronary CTA based on symptoms or pre-existing cardiovascular risk factors. The low prevalence of smoking and diabetes may explain the lack of a statistically significant association between these known cardiovascular risk factors and CAD in our study population. Further, the low prevalence of these risk factors precluded calculation of odds ratios of BAC related to these risk factors.

The relationship between BAC and cardiovascular morbidity remains obscure. BAC is typically characterized as Monckeberg medial calcific sclerosis, which affects the media and is distinct from the calcification and thickening of the arterial intima that occurs in medium to large sized arteries and is commonly characterized as ‘typical’ atherosclerosis [33]. In addition to being related to aging [34], medial sclerosis is known to be accelerated by chronic diseases such as diabetes and hypertension [35], although this relationship has been incompletely investigated in studies comparing BAC with diabetes and hypertension [8, 27]. In general presence of medial sclerosis has been associated with an increased risk of complications of diabetes mellitus [36].

The relative strength of the association between BAC and cardiovascular morbidity and mortality versus that of BAC and CAD as defined by luminal stenosis, e.g. on conventional catheter coronary angiography, may be due to the well documented differences between men and women in morphologic expression of coronary artery disease. The Women’s Ischemic Syndrome Evaluation Study found that the value of angiography in predicting myocardial infarction was substantially decreased in women compared to men [2, 3, 37]. These findings suggest that in women, functional and microvascular abnormalities in the cardiovascular circulation may exist long before luminal stenotic disease of coronary arteries is depicted on catheter angiography.

This study’s findings corroborate additional recent research demonstrating that traditional techniques for assessing CAD have been less effective in women. For example, exercise ECG—long the cornerstone of diagnostic techniques for assessing CAD in men—appears less accurate for diagnosing ischemic heart disease in women [38]. Furthermore, the Framingham Study has demonstrated that chest pain, a strong indicator of cardiac disease in men, is significantly less reliable in women [39].

Considering the current controversy over changing recommendations for screening mammography, public uncertainty regarding the safety and efficacy of screening mammography, the declining rates of screening mammography utilization, and the lack of a reliable screening instrument for cardiovascular disease in women, the use of BAC as a potential marker of CAD can have a broad impact on public health. This additional vascular information yielded by screening mammography may therefore enhance its role as a screening and contribute to the diagnosis of not only one but two of the most fatal diseases in women, breast cancer and coronary artery disease.

To our knowledge no prior study has attempted to characterize the relationship between BAC and CAD as determined by coronary CTA. The strong association between BAC and CAD by CTA suggests that BAC may serve as an independent predictor for cardiovascular morbidity and potentially mortality. Clearly, additional studies with larger patient populations are warranted to better define the diagnostic value of mammographically detected BAC.

## Conclusions

BAC on mammography appears associated with the presence of CAD as determined by coronary CTA. Given the tendency to underdiagnose cardiovascular disease in women and the lack of a reliable screening instrument, the presence of BAC on screening mammography may indicate an increased risk for cardiovascular disease in the appropriate clinical context.
